# Computational Frameworks, System-Level Platforms, and Emerging Trends in DNA Data Storage

**DOI:** 10.34133/csbj.0254

**Published:** 2026-11-10

**Authors:** Jun Luo, Ziqi Meng, Xinyao Tai, Chunxia Ge

**Affiliations:** ^1^ Second Clinical Medical College, Shandong Medical and Pharmaceutical University, Yantai City 264003, China.; ^2^ Shandong Key Lab of Complex Medical Intelligence and Aging, Shandong Medical and Pharmaceutical University, Yantai City 264003, China.; ^3^ School of Pharmacy, Shandong Medical and Pharmaceutical University, Yantai City 264003, China.; ^4^ School of Health Management, Shandong Medical and Pharmaceutical University, Yantai City 264003, China.

## Abstract

DNA storage has emerged as a promising approach for long-term digital information preservation owing to its ultrahigh storage density, long-term stability, and potential for passive long-term preservation without continuous electrical power under appropriate storage conditions. Recent advances in DNA synthesis, sequencing, and molecular manipulation technologies have accelerated the development of the field, broadening the scope of research from sequence-based encoding to biochemical channel modeling, system implementation, structured DNA storage, and information management. However, existing studies are often focused on individual components of DNA storage systems, and the relationships among these components remain insufficiently characterized from a computational and system-level perspective. This structured narrative review examines selected representative studies based on their technical importance and relevance to a computational and system-level analytical framework for DNA storage. We first outline the development of sequence-based and non-sequence-based DNA storage technologies, highlighting the expansion of information representation beyond nucleotide sequences to encompass multidimensional molecular structures and states. We then examine representative computational tools and system-level platforms, including coding toolchains, biochemical channel modeling, automated storage platforms, and user-oriented information management systems. We present an analytical framework comprising 5 interconnected functional layers: information representation, encoding and decoding, biochemical channel, system implementation, and access and management. Using this framework, we analyze the relationships among existing technologies, current technical challenges, and emerging research directions. DNA storage is evolving from a high-density storage technology toward an integrated molecular information system that supports information representation, storage, retrieval, and management. Further progress will require coordinated advances in multidimensional information representation, content-based querying, cross-layer system optimization, and interdisciplinary research to support the development of practical DNA-based information systems.

## Introduction

With the rapid advancement of the digital era, the volume of global data has been increasing exponentially. In particular, the continuous accumulation of images, videos, scientific datasets, and artificial intelligence training data poses substantial challenges for conventional magnetic and semiconductor storage technologies in terms of storage capacity, energy consumption, and long-term data preservation [1–3]. Consequently, the development of novel storage media with a high storage density, low energy requirements, and long-term stability has become a major research focus in the field of information storage.

As the natural carrier of genetic information, DNA possesses exceptional storage density and long-term stability, while its molecular information can be preserved without continuous electrical power under appropriate storage conditions, making it one of the most promising candidates for next-generation data storage [4–6]. However, passive preservation does not necessarily imply low energy demand for the DNA storage system as a whole. The overall energy demand depends on the energy and material inputs associated with data writing, storage, and readout [7,8]. The theoretical storage capacity of DNA is 455 EB per gram [4]. Furthermore, the estimated half-life of 150-nt DNA fragments is approximately 38,000 years under specific preservation conditions, highlighting DNA’s potential for ultralong-term information storage [9]. Recent advances in DNA synthesis, sequencing, and molecular manipulation have supported the development of diverse DNA storage approaches, including fountain-code-based storage, optimized error-correcting schemes, random-access strategies, and structured DNA storage. These advances have expanded the field into a broad research area encompassing encoding design, error control, system implementation, and data access [10–13].

Current research has primarily focused on encoding optimization, biochemical constraint modeling, and experimental validation, leading to substantial progress in each of these areas [14–16]. However, most studies address only specific components of the DNA storage workflow, while systematic analyses covering the entire information-processing pipeline remain limited. At the same time, emerging technologies such as structured DNA storage [17,18], epigenetic data storage [19,20], and automated experimental platforms have broadened DNA storage research to encompass information representation, semantic access, and system management alongside storage-density optimization and data recovery [21,22].

Against this background, this review examines DNA information storage from a systems perspective. We first summarize the underlying technologies and the historical development of DNA storage. We then examine representative system platforms, channel modeling tools, and encoding toolchains, highlighting their functional roles and methodological characteristics. Building upon this analysis, we propose a cross-layer framework that organizes existing research around information representation, encoding and decoding, biochemical channel, system implementation, and access and management. Finally, we discuss emerging trends and outline future research directions and engineering opportunities.

This study used a structured search and screening strategy for a narrative review of the relevant literature. The search was conducted across 5 databases Web of Science, Scopus, PubMed, IEEE Xplore, and Google Scholar, and included publications available from their respective coverage start dates through 2026 June 1. The core search keywords included “DNA storage”, “DNA data storage”, “encoding”, “error correction”, “simulation”, “retrieval”, “toolkit”, and “platform”. These terms were combined and adapted to the search syntax of each source.

Records retrieved from the search were first deduplicated, and the remaining records were then screened based on their titles and abstracts. The full texts were then assessed for eligibility. The inclusion criteria were as follows: (a) studies directly related to digital DNA information storage; (b) studies addressing at least one aspect of DNA storage, including information encoding, error correction, simulation, retrieval, computational tools, software toolkits, experimental platforms, or system implementation; and (c) studies providing sufficient information about their technical methods, tool functionalities, or system implementation for analysis. The exclusion criteria were as follows: (a) studies not directly related to digital DNA information storage; (b) studies focusing solely on DNA molecular biology, medical applications, or other applications without involvement in digital information storage; (c) literature lacking technical or system information relevant to the analytical framework of this study; and (d) duplicate publications or studies for which sufficient full-text information was unavailable for evaluation.

After the literature search and screening were completed, this paper does not discuss all included studies at the same level of detail. Instead, representative tools, platforms, and methods from different research directions are selected for focused analysis according to their functions, technical characteristics, methodological contributions, and roles in the DNA storage workflow. Accordingly, the tools and platforms discussed in Computational Tools and System-Level Platforms for DNA Data Storage and Computational Framework and Key Challenges in DNA Data Storage are intended to cover the key computational and system-level components of DNA storage, rather than to provide an exhaustive catalog of all existing tools and platforms. This selection is intended to provide broad coverage of the field while minimizing redundant descriptions.

## Overview of DNA Data Storage Technologies

Based on how information is represented, DNA data storage can be broadly classified into 2 categories: sequence-based [23] and non-sequence-based approaches. These 2 paradigms differ not only in how information is encoded but also in the broader research directions they represent. Together, they illustrate the expansion of DNA storage research from sequence-oriented encoding toward multidimensional molecular information representation.

### Classification of DNA data storage

Sequence-based DNA storage is currently the most mature and widely adopted approach. Digital information is encoded as DNA sequences composed of the 4 DNA nucleotides (A, T, C, and G) according to predefined mapping rules, with DNA synthesis and sequencing serving as the writing and readout processes, respectively. To improve robustness against insertion, deletion, and substitution errors, error-correcting schemes such as fountain codes [11], Reed–Solomon codes [24,25], and convolutional codes [26] have been incorporated into DNA storage systems. Owing to their compatibility with existing high-throughput sequencing technologies, sequence-based approaches are promising candidates for archival storage of cold data at large scales.

Meanwhile, coding strategies in DNA storage have increasingly moved beyond general-purpose error-correcting codes to include specialized codes tailored to specific molecular storage channels. As DNA storage typically preserves data in the form of an unordered set of molecules rather than a single continuous sequence, coding schemes must address not only intrasequence insertion, deletion, and substitution errors but also set-level errors such as molecule loss. In response to this characteristic, existing studies have modeled DNA storage as a set-based channel and incorporated multiple error types, including strand loss, symbol substitutions, and insertions/deletions, into a unified coding framework [27,28]. Building on this work, related studies have accounted for the random replication and sampling processes of DNA molecules by extending the storage channel model to incorporate a multidraw insertion–deletion–substitution model. They have also improved data recovery through the concatenation of outer codes, index codes, and synchronization codes [29]. Accordingly, the coding challenges in DNA storage have expanded from symbol-level error correction to molecular-set-level problems involving molecule loss, sampling, and synchronization.

Sequence-based information representation is also not limited to deterministic single-nucleotide symbols. Composite DNA letters and probabilistic DNA representations further expand the ways in which information can be represented within the sequence space, motivating a shift in coding models from discrete-symbol error correction toward methods that account for probabilistic uncertainty. Instead of using individual DNA nucleotides as basic information units, composite DNA alphabets encode information through probability distributions of base-composition ratios across a population of DNA molecules, thereby expanding the effective alphabet within the sequence space. Accordingly, the coding design for these representations needs to account for both the inherent uncertainty of information representation and the probabilistic bias introduced during the storage process [30]. For information described by such probability vectors, researchers have developed specialized error-correction coding models to address bounded-magnitude errors in probability vectors [31]. Meanwhile, as DNA storage channel models have become more refined, coding design has increasingly incorporated molecular sampling, nonuniform inter-strand insertion/deletion/substitution errors, and indexing and synchronization procedures into an end-to-end framework [32]. DNA storage coding has expanded from conventional error correction for discrete nucleotide sequences to jointly addressing probability distributions and perturbations introduced by molecular storage channels. Coding designs are increasingly channel aware and tailored to specific information representations, sampling processes, and error characteristics.

These theoretical and methodological advances have been demonstrated in DNA storage prototype systems. Organick *et al.* encoded 35 independent files totaling over 200 MB of data and synthesized 13 million 150-nt oligonucleotides. After accounting for the overhead from primers, indices, and fountain-code redundancy, the reported net information density was 1.1 bit/nt. All stored files were reported to be recovered individually without errors at a median sequencing coverage of approximately 5 reads per sequence, with individual files ranging from 4 to 14 reads per sequence [12,33].

Despite these advances, sequence-based DNA storage remains fundamentally sequence centric, with its performance highly dependent on synthesis accuracy, sequencing coverage, and error-correction capability [5,23]. Conventional DNA storage channel models generally adopt simplified stochastic error assumptions and characterize sequencing using average error rates [5]. DNA-specific encoding and decoding models have increasingly incorporated factors such as random sampling, molecule loss, and strand-level insertions, deletions, and substitutions [29,32]. Nevertheless, in practical DNA storage workflows, sequence characteristics such as GC content, together with synthesis and polymerase chain reaction (PCR) amplification biases, can lead to uneven sequencing coverage and systematic errors [34,35]. Moreover, sequence constraints such as GC-content limits and homopolymer restrictions increase the complexity of sequence design and can complicate decoding when insertion and deletion errors occur [36,37].

To move beyond a storage paradigm in which information is represented solely by deterministic nucleotide sequences, researchers have increasingly explored non-sequence-based approaches to DNA information storage. These approaches exploit the spatial conformation, topological configuration, or epigenetic modifications of DNA molecules to encode information, thereby introducing additional information dimensions beyond the nucleotide sequence.

Some studies have also used DNA in living cells as an information carrier, encoding digital information into bacterial genomes and maintaining and propagating it through cellular replication and inheritance. Existing studies have employed CRISPR–Cas systems to encode digital information such as images and videos into living bacterial genomes and have explored approaches to digital information recording based on CRISPR arrays [38,39]. Unlike storage approaches based on DNA synthesis and *ex vivo* preservation, *in vivo* DNA storage incorporates living cells and their genetic processes into the storage system. This enables DNA information to be maintained and replicated through cellular processes, representing a distinct implementation pathway for DNA information storage. However, this approach is also constrained by factors including cell growth, mutation accumulation, carrier capacity, and information readout [5,39].

### Development of sequence-based DNA data storage

The evolution of sequence-based DNA data storage (Fig. 1) can be viewed as a continuous effort to balance 3 fundamental objectives: coding efficiency, channel reliability, and biochemical feasibility. With advances in DNA synthesis, amplification, and sequencing technologies, research has increasingly focused on coding efficiency, error resilience, and system-level optimization, following early demonstrations of storage feasibility. Accordingly, the development of sequence-based DNA storage can be broadly divided into 3 stages: proof of concept, encoding optimization, and system-oriented design.

**Fig. 1.**
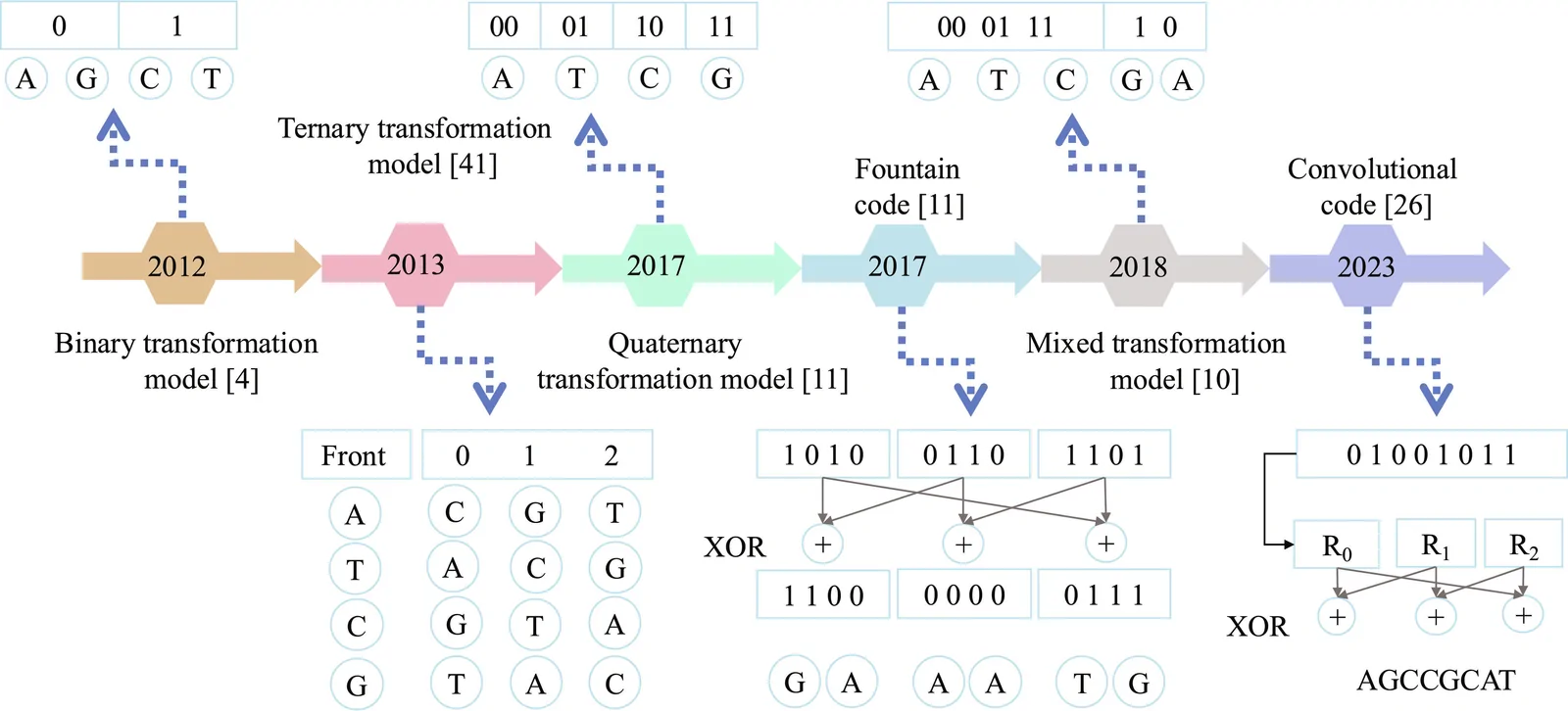
Evolution of sequence-based DNA encoding strategies. Representative sequence-based DNA encoding strategies developed from binary, ternary, and quaternary transformation models to more advanced coding schemes, including DNA Fountain, mixed transformation models, and convolutional encoding. This progression reflects a move from simple sequence mapping toward robust, high-density, and error-resilient information encoding.

The initial stage focused on demonstrating that digital information could be reliably encoded into DNA and accurately recovered [40]. Using a simple binary mapping strategy, the Church group [4] converted digital data into DNA sequences, establishing the work as one of the foundational studies in synthetic DNA digital information storage. Since the main objective at this stage was to establish storage feasibility, encoding strategies focused mainly on mapping digital information to DNA sequences, while storage density, biochemical constraints, and error correction received less attention. Consequently, the effective information density and overall storage efficiency were relatively limited.

As storage capacity increased, research shifted toward improving both information density and data recovery performance [11,12,41]. To increase the information carried by each nucleotide, ternary, quaternary, and other coding schemes were proposed to better exploit the encoding capacity of DNA sequences and achieve a higher information density [11,41]. At the same time, DNA storage was increasingly modeled as a noisy biochemical communication channel [5,23], with data recovery affected by synthesis errors, PCR amplification bias, and sequencing noise. As research progressed, methods such as fountain codes, Reed–Solomon codes, and convolutional codes were introduced to improve data recovery in the presence of insertion, deletion, and substitution errors [11,41,42]. Among these developments, DNA Fountain demonstrated high coding efficiency while approaching the theoretical limit of 2 bits/nt, representing an important advance in the pursuit of high-density and reliable DNA storage. During this period, research increasingly focused on how to store information reliably, beyond demonstrating storage feasibility alone. This stage also advanced research on noisy biochemical channel modeling and constrained coding beyond simple data mapping, contributing to the development of theoretical frameworks for DNA storage.

For DNA storage using deterministic encoding with only the 4 nucleotide bases A, C, G, and T, each encoding position has 4 possible symbols, corresponding to a theoretical information-capacity limit of 2 bits/nt. However, the effective information density achieved in practice, after accounting for addressing, error correction, and biochemical constraints, is generally lower than this upper limit. DNA Fountain has been reported to achieve approximately 1.57 bits/nt. By contrast, another type of DNA storage scheme does not restrict each encoding position to a single deterministic nucleotide base. Instead, it encodes information using the relative proportions of the 4 bases—A, C, G, and T—in a molecular population, thereby expanding the distinguishable symbol space and potentially enabling information capacities above the 2 bits/nt limit of deterministic 4-base encoding.

Advances in high-density encoding have enabled composite DNA storage (Fig. 2) to explore information capacities beyond the theoretical 2 bits/nt limit of traditional 4-base deterministic encoding. Composite DNA letters are defined by the relative proportions of A, C, G, and T across the molecular population, thereby expanding the information-representation space without requiring longer individual DNA strands. Anavy *et al.* demonstrated the feasibility of this approach. In an experiment involving approximately 6.4 MB of data, the method achieved a coding efficiency of 1.96 bits/synthesis cycle and reduced the number of synthesis cycles required per unit of data by approximately 20%. With further expansion of the composite symbol space, small-scale experiments achieved a coding efficiency of 4.29 bits/synthesis cycle, while simulations indicated a potential efficiency of approximately 6.4 bits/synthesis cycle [30]. Building on this foundation, researchers have also explored higher-dimensional encoding structures formed by combining molecular units, alongside efforts to expand the composite symbol space. Yan *et al.* [43] proposed composite motifs, whereas Preuss *et al.* [44] employed shortmer combinatorial encoding to further expand the coding space and reduce synthesis overhead.

**Fig. 2.**
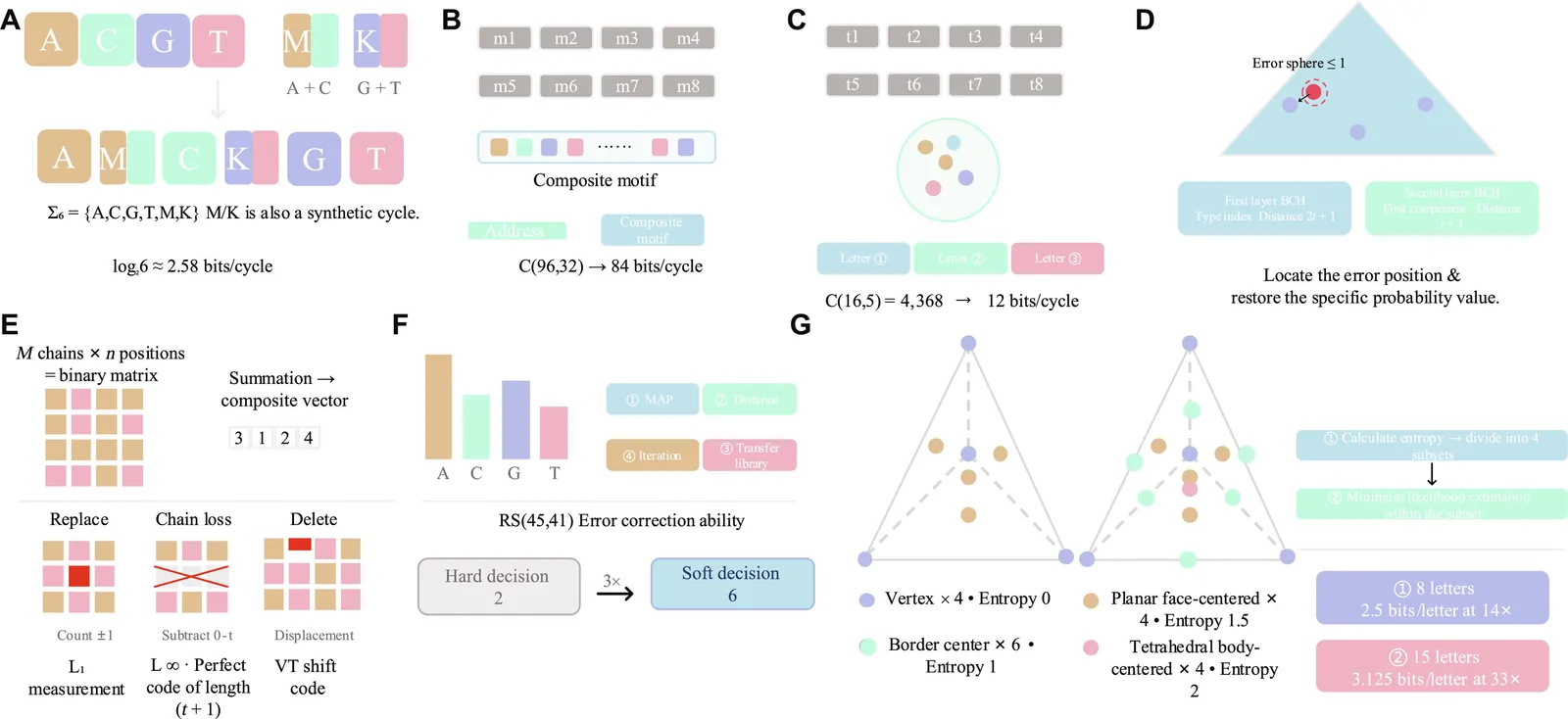
Development of encoding and decoding strategies for composite DNA storage. (A) Construction of a composite alphabet space based on the proportional distribution of A, C, G, and T in a population of DNA molecules. (B) Enhancement of the information representation capacity per synthesis cycle by expanding the number of composite letters. (C) Alternative strategies for expanding the encoding space, including enzymatically ligated composite motifs and shortmer combinatorial encoding based on distinguishable DNA shortmers. (D) Information representation, channel modeling, and readout processes of composite DNA storage. (E) Channel models and error-correction strategies developed for different error types, including probabilistic amplitude errors, substitutions, deletions, and strand loss. (F) Improvement of composite-letter identification and data recovery capability through soft decision based on the base-frequency distribution obtained from sequencing. (G) Construction of a composite-letter constellation by DNA diamond via geometric constraints, realizing the co-design of encoding space and reliable readout.

However, expanding the composite-letter space not only improves coding efficiency but also changes the information representation mode and channel characteristics of DNA storage. Because composite letters are defined by the base-composition ratios within the molecular population, limited sequencing depth introduces sampling fluctuations, while synthesis and sequencing errors further distort the observed proportions. Consequently, composite-letter discrimination relies on inference from probability distributions rather than deterministic recognition of individual bases. Accordingly, subsequent research has increasingly addressed not only coding-space expansion but also the accuracy of composite-letter discrimination and the reliability of data recovery. One line of research focuses on channel modeling and error-correction methods in composite DNA storage. Zhang and Wang [31] developed error-correction models specifically for limited-magnitude probability errors on probability vectors, whereas Walter *et al.* [45] further considered the error types arising in DNA storage scenarios, including substitutions, complete strand loss, and deletions.

Another line of research directly optimizes the readout process by leveraging the base-frequency distributions estimated from sequencing data for composite letters. Xu *et al.* [46] employed soft-decision decoding based on sequencing-derived base frequencies, improving the accuracy of composite-letter discrimination while reducing the required sequencing depth by approximately 50%. The 2 types of methods exhibit distinct optimization strategies: the former focuses on characterizing probabilistic errors and complex error patterns in composite DNA storage, thereby improving the reliability of error-correction codes; the latter leverages probabilistic information in the observed data to improve composite-letter discrimination. Decoding strategies for composite DNA storage are gradually evolving from traditional hard-decision approaches to channel-aware soft-decision decoding. Building on these advances, researchers have increasingly explored the co-design of symbol spaces, molecular structures, and readout mechanisms, in addition to optimizing coding and recovery. Ge *et al.* proposed DNA diamond, a scheme that maps composite letters into a geometrically constrained tetrahedral space to form a decomposable letter constellation. At a scale of 10,000 composite strands, the 8-letter system achieved a payload density of 2.5 bits/letter with error-free recovery at 14× sequencing coverage, whereas the 15-letter system achieved 3.125 bits/letter with error-free recovery at 33× sequencing coverage [47]. Overall, the field has expanded its focus beyond coding-space enlargement to include the joint optimization of information representation, probabilistic channel modeling, and reliable readout. However, further increasing the capacity of composite encoding can increase molecular compositional diversity, raise sequencing-coverage requirements, and complicate composite-letter discrimination. How to strike a balance among information density, sequencing cost, and decoding complexity remains a key challenge for advancing composite DNA storage.

In recent years, the continuous growth of storage capacity and application demands has driven DNA storage research beyond coding optimization toward system-level design [48]. Researchers have increasingly considered coding efficiency, biochemical constraints, error propagation, random access, and read–write workflow optimization within a unified framework, using channel simulation platforms and performance evaluation tools for system modeling and analysis [5,49–51]. Meanwhile, emerging paradigms such as non-sequence-based DNA storage exploit nonsequence features—including spatial conformations, topological configurations, and epigenetic modifications—as additional information-carrying dimensions [52,53]. Greater attention has been devoted to error propagation in realistic biochemical channels, workflow coordination, and engineering implementation, marking a shift from coding theory-driven research toward a systems engineering paradigm.

### Development of non-sequence-based DNA data storage

Non-sequence-based DNA storage emphasizes multidimensional information representation, molecular state regulation, and programmable functionality, thereby providing a basis for developing next-generation molecular information systems with enhanced functional scalability [54,55]. The development of this field is summarized in Fig. 3.

**Fig. 3.**
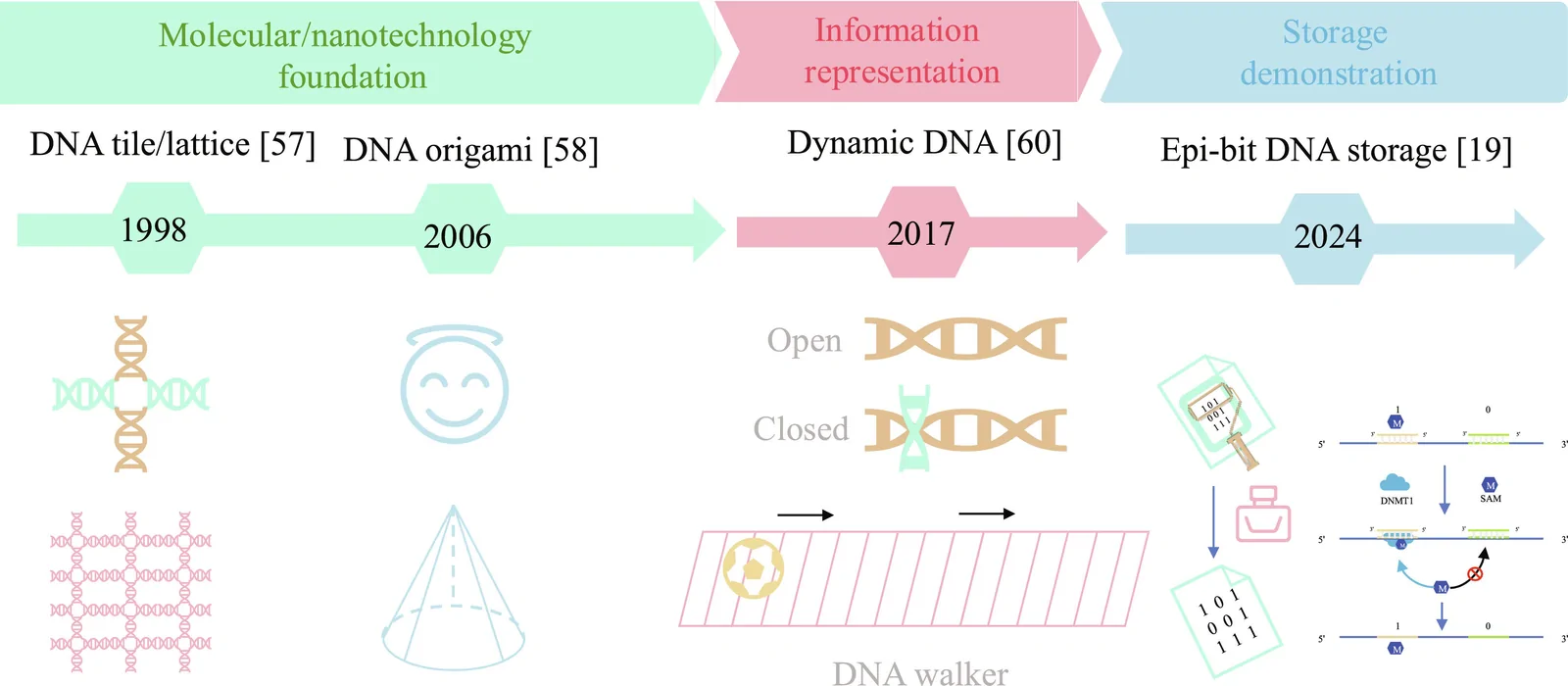
Evolution of non-sequence-based DNA encoding. Representative developments include DNA tile/lattice assemblies, DNA origami, dynamic DNA nanostructures, and recent DNA structural storage systems. This trajectory illustrates the progressive expansion of DNA-based information representation from nucleotide sequences toward spatial structures, molecular states, and programmable molecular architectures, while the demonstrated functions range from structural assembly and molecular actuation to information storage and retrieval.

This study indicates that non-sequence-based DNA storage has primarily advanced along 3 directions: molecular structure encoding, dynamic state encoding, and epigenetic modification encoding [56]. Studies including DNA origami, 3-dimensional DNA architectures, and DNA bricks have demonstrated that complex DNA nanostructures can be precisely designed and assembled, thereby providing the basis for representing information through spatial conformation [57–60]. Technologies and mechanisms such as DNA nicking, strand displacement reactions, and dynamic DNA devices have further extended information encoding to the structural state level. Specifically, 2-dimensional DNA storage systems utilize nicks on the DNA backbone to record additional information and combine this approach with machine learning to support data reconstruction and rewriting [55,61]. DNA origami has also been applied to construct structured storage units capable of encoding and reading data, extending its applications from structural design to information storage [62]. More recently, epigenetic strategies such as DNA methylation have enabled additional molecular state information to be recorded without altering the underlying nucleotide sequence [19]. Together, these approaches provide different mechanisms for encoding information in molecular structures and states, with demonstrated capabilities varying across writing, readout, reconstruction, and rewriting.

The forms of information represented in DNA storage have gradually expanded from linear nucleotide sequences to structured representations with spatial relationships, topological features, and molecular states [18,19,55]. Conventional DNA storage has primarily focused on sequence mapping, constrained coding, and error correction, whereas non-sequence-based approaches introduce additional challenges involving structural design, assembly optimization, state regulation, and structural decoding [56,63]. These approaches require computational representations capable of capturing graph structures, topological relationships, and molecular states [18]. Such representations can support structure-level information processing, but they also increase the complexity of system design, structural decoding, and data access. Increasing the dimensions of information representation does not necessarily lead to a proportional increase in effective storage capacity. Previous studies have shown that structural information representation typically relies on additional DNA components and assembly processes. Information representation based on DNA secondary structures may require additional nucleotides to form the corresponding structures [23,64]. By contrast, information storage based on molecular modification states requires corresponding chemical or enzymatic writing and readout [19]. Thus, although nonsequence information carriers introduce additional information dimensions beyond the base sequence, implementing these dimensions incurs costs associated with molecular construction, assembly, writing, and readout. Therefore, the capacity of nonsequence DNA storage should be evaluated separately from capacity estimates based solely on sequence length or theoretical state space while accounting for the molecular resources and operational costs associated with implementing additional information dimensions.

Relevant studies differ in technical positioning and functional scope and can be grouped into 3 categories. The first category consists of basic research on DNA nanostructures and molecular devices, including DNA origami, DNA bricks, and dynamic DNA nanotechnology. These studies mainly focus on programmable DNA self-assembly, spatial structure construction, and molecular state regulation, thereby providing a foundation for structured information storage [58,59,61]. The second category covers research on structured DNA information storage and molecular-level manipulation, including 2-dimensional DNA storage and DNA-origami-linked storage. These approaches have demonstrated capabilities such as information encoding, reconstruction, and limited dynamic access [18,55]. The third category comprises DNA data storage systems that implement an end-to-end storage workflow. For example, epigenetic-modification-based storage supports information encoding, molecular writing, readout, and recovery [19]. Therefore, although DNA nanostructure-related research shares a common molecular basis, there remain marked differences in information-carrying modes, access mechanisms, and system functional scope.

Based on the differences described above, this study uses 2 operational criteria for including nonsequence DNA information systems in direct comparison: (a) the presence of clearly identifiable nonsequence information-carrying states and (b) the experimental demonstration of at least one information-related operation. Here, information-related operations include writing, reading, recovery, or rewriting. Studies primarily focused on structural assembly, molecular computation, indexing, or molecular actuation, but which do not implement digital information storage and recovery, are discussed only as relevant technical foundations and are not included in the direct comparison of storage performance. For hybrid systems that simultaneously rely on base sequences and nonsequence states, the information-carrying dimensions and functions are recorded separately rather than the systems being classified as purely nonsequence storage. Based on these criteria, this study classifies the representative studies in Table S1 into 2 groups according to their information-bearing states and experimentally validated functions: foundational molecular technologies and experimentally demonstrated digital information storage systems. Only the latter are included in the quantitative comparison of storage performance.

These richer representations raise new system-design challenges, including molecular architecture design, assembly control, error modeling, and information decoding. Currently, non-sequence-based DNA storage remains in the exploratory stage with respect to read–write efficiency, system reliability, and standardized performance evaluation [23,56,65], which continues to constrain its practical application. The demonstrated ability of some systems to support molecular-level access or manipulation should not be interpreted as a general advantage in random access across non-sequence-based DNA storage. When assessing emerging directions such as molecular computation, content-based querying, and integrated information management systems, the computational and system requirements imposed by structured information representation must be taken into full account. Considerable variation exists across different DNA storage strategies in terms of information density, coding redundancy, data recovery performance, and experimental scale.

### Technical challenges in DNA data storage

Regardless of whether DNA data storage is sequence-based or non-sequence-based, its fundamental objective is to encode, preserve, and recover digital information using DNA molecules. A typical DNA storage system consists of 5 key stages: data encoding, DNA synthesis, storage, sequencing-based readout, and decoding. Among these stages, encoding determines how information is represented; synthesis and sequencing serve as the write and readout processes, respectively; and decoding reconstructs the original digital data. These stages are tightly interconnected and collectively determine the storage efficiency, reliability, and overall cost of the system [66].

The primary challenges facing DNA data storage arise from the coupling between biochemical processes and information processing. On the one hand, the high cost of DNA synthesis and sequencing, together with limited writing speed and throughput, continues to constrain large-scale practical deployment. On the other hand, substitution, insertion, and deletion errors introduced during synthesis, amplification, and sequencing directly affect data recovery, while sequence-related and structural features, such as GC-content bias, homopolymer regions, and secondary structures, contribute to nonuniform error distributions [5]. Consequently, developing accurate channel models and improving system robustness have become central objectives in DNA data storage research [11,41,51]. The low efficiency and nonuniformity of biochemical reactions, together with the time required for synthesis, amplification, manipulation, and sequencing, remain key factors affecting the feasibility and large-scale development of DNA storage systems [53].

These factors collectively limit the extent to which theoretical encoding density can be translated into practical storage performance. First, errors that accumulate during the DNA synthesis process can reduce the yield of full-length DNA strands, thereby limiting the effective storage capacity achievable in practice. For stepwise DNA synthesis, the theoretical yield of full-length products can be expressed as Eq. (1):

$$Y=\eta^{L-1}$$ (1)

This equation provides an idealized estimate based on the assumption that coupling efficiency is approximately constant and independent at each synthesis step. It does not represent a comprehensive model of actual oligonucleotide yield. Taking a 200-nt sequence as an example, when the per-step coupling efficiency increases from 99.3% to 99.9%, the theoretical proportion of full-length products rises from 24.7% to 81.9%, illustrating how sequence length and per-step synthesis efficiency jointly affect full-length product yield [67]. In practice, oligonucleotide yield is also affected by sequence-dependent effects, purification procedures, truncation products, depurination, the synthesis platform, and other chemistry-specific factors.

Second, coding redundancy further creates a trade-off between information density and reliability. Early systems mainly relied on duplication, overlap, and checksum information to improve recovery capability. Subsequent approaches introduced more advanced channel-oriented coding strategies, such as DNA Fountain. For example, DNA Fountain achieves error-free recovery of 2.14 × 10^6^ bytes of data at an effective information density of 1.57 bits/nt [11]. In general, increasing coding redundancy reduces the proportion of the synthesized data devoted to the effective payload.

Practical systems are also constrained by molecular bias during synthesis, amplification, and sequencing. A quantitative study of 1,536,168 DNA sequences showed variation in sequence abundance and changes in population fractions, illustrating how PCR amplification can introduce nonuniformities that deviate from the uniform molecular population assumed in idealized coding models [34]. Therefore, DNA storage performance should not be evaluated solely on the basis of theoretical information density; instead, the evaluation should account for coding redundancy, synthesis fidelity, molecular bias, sequencing coverage, and final recovery performance.

Efficient access to and utilization of stored information has become an increasingly prominent requirement as DNA storage systems scale up. Existing DNA storage systems primarily rely on PCR primers to achieve file-level random access, whereas direct retrieval based on local content, structural features, or semantic information remains limited. Early random access in DNA storage mainly relied on predefined primers or barcodes to locate target DNA molecules using sequence-based addresses [68]. As access demands expand from target localization to selective manipulation and data querying, random-access mechanisms have expanded beyond sequence-based addressing to include physical positions and data content. Electric-field-guided DNA access spatially selects target DNA pools using microelectrode arrays and performs primer-mediated DNA extension directly at the selected positions, thus enabling selective access to specific DNA pools without PCR amplification [69]. Existing work has started to explore content-based querying modes, including content-based filter queries [70] and molecular-level similarity search [71]. However, these studies demonstrate task-specific content-based querying and similarity search but do not yet establish a general-purpose semantic retrieval system for DNA data storage. Building on existing studies, future research could explore the integration of vector representations, semantic indexing, and hierarchical information organization to support more flexible access at the content, structural, and semantic levels. Table S2 summarizes the reported characteristics and performance metrics of representative DNA storage approaches, highlighting the diversity of experimental conditions and evaluation criteria across studies. However, reported performance varies substantially among studies, partly because of differences in encoding schemes, experimental conditions, sequencing coverage, and evaluation criteria. Meanwhile, engineering metrics such as overall cost, cost per byte, practical data-writing and readout speeds, and error rate are still incompletely and inconsistently reported across existing studies, limiting direct quantitative comparison among different DNA storage systems. Recent work on standardized benchmarking of DNA storage codecs has demonstrated the importance of standardizing evaluation conditions, including coding rate, sequence length, physical redundancy, sequencing depth, and error conditions, to enable fair performance comparisons among codecs [72].

## Computational Tools and System-Level Platforms for DNA Data Storage

To address challenges in encoding design, error correction, data recovery, and system implementation, a wide range of computational tools and software platforms have been developed for DNA data storage [5]. However, these tools target different levels of the storage workflow. Some are designed to improve coding efficiency and error-correction performance, whereas others focus on modeling biochemical processes, such as PCR amplification bias, sequencing errors, and coverage variation. In addition, several platforms provide end-to-end solutions for automated data writing, random access, and experimental workflow validation. Consequently, classifications based solely on algorithmic characteristics or experimental procedures do not adequately capture the functional roles of these tools or their relationships within the overall DNA storage system.

The associated computational tools serve different functional levels within the DNA storage workflow [51]. Based on their methodological roles, existing tools can be broadly categorized into 4 groups: system-level platforms, encoding standards and channel modeling tools, general-purpose encoding and decoding toolchains, and user-oriented storage and information management platforms. Among them, system-level platforms are primarily intended for end-to-end workflow validation and engineering implementation. Encoding standards and channel modeling tools address problems related to information representation and biochemical channels, respectively, whereas general-purpose toolchains focus on data encoding, error recovery, and performance evaluation. Based on this classification framework, this paper selects representative tools and systems for discussion according to their functional positioning in the DNA storage workflow and representative technical directions. The specific platforms and the rationale for their selection are outlined in Table 1; this paper does not intend to provide a performance ranking under uniform test conditions.

**Table 1.** Representative computational methods and platforms for DNA data storage

Technical category	Representative platform	Core functions	Rationale for selection
System-level platforms	DNA-DISK	Integration of DNA synthesis, storage, sequencing, and automated workflows	Illustrates the progression from discrete experimental steps to automated, end-to-end system integration
Encoding standards and channel modeling	JPEG DNA	DNA image encoding and standardization-oriented information representation	Illustrates data-type-specific standardized information representation for image data
	MESA	Error simulation for DNA synthesis, PCR, storage, and sequencing	A classic approach for unified channel modeling and error simulation across multistage biochemical processes in DNA storage
Encoding-channel co-design	DNA Fountain	Fountain-code encoding adapted to DNA biochemical constraints	A representative and influential early example of the encoding-channel co-design approach
	DNA Storage Toolkit	Encoding, simulation, clustering, reconstruction, and decoding	Represents the direction of DNA storage evolving from single encoding algorithms to modular, end-to-end toolchains
	DNA-Chain	Image structural representation, encoding, and error correction	Represents the recent trend of DNA storage placing increasing emphasis on data structure representation, content organization, and information utilization
User-end storage and information applications	iDNAdrive	Data storage, access, and information management	Represents the direction of DNA storage expanding from low-level encoding and experimental validation to user-side data storage, access, and information management applications

PCR, polymerase chain reaction

### Engineering of system-level platforms for DNA data storage

Unlike conventional DNA storage research, which primarily focuses on coding efficiency and error-correction performance, system-level platforms place greater emphasis on validating the practical feasibility of DNA storage under wet-lab conditions. Early studies primarily focused on automating and validating DNA storage workflows. Takahashi *et al.* [73] integrated data writing and data recovery into an automated experimental platform, demonstrating the feasibility of end-to-end automated operation of DNA storage systems. Li *et al.* [74] proposed the DNA-DISK platform, which integrated automated control, DNA writing, readout, and recovery processes, advancing the integration of end-to-end DNA storage systems and providing a technical basis for their development toward practical applications (as shown in Table 2). Microfluidic platforms have also been explored for the active assembly and processing of DNA data within molecular pools, extending their potential applications beyond workflow automation. For example, Yu *et al.* combined a droplet-controlled fluidics system with splint ligation to enable programmable assembly of DNA fragments. By processing encoded sequences in both serial and parallel modes, the system constructed defined DNA pools with PCR-based random-access capability [75].

**Table 2.** Key technical characteristics of the DNA-DISK system-level platform

Category	DNA-DISK [74]
Positioning	Automated prototype DNA data storage system
Core technologies	• Digital microfluidics (DMF) • Enzymatic single-base DNA synthesis • Pyrosequencing
Key innovation	Integration of encoding, synthesis, and sequencing on a single microfluidic chip, enabling end-to-end automated DNA data storage
Data processing	Simple binary encoding and data fragmentation for small-scale writing demonstrations
Application	Validation of automated DNA data storage workflows

The value of system-level platforms lies in evaluating DNA storage workflows under realistic biochemical conditions, thereby revealing engineering constraints that are difficult to capture by theoretical models alone. Factors such as synthesis efficiency, platform-specific operations (e.g., droplet scheduling), PCR amplification bias, and the degree of system integration all affect the final data recovery performance. Accordingly, DNA storage performance depends on the interaction between encoding strategies, biochemical channel characteristics, and hardware platforms. Compared with algorithm-level evaluations, system-level platforms provide a more comprehensive assessment of the practical performance of DNA storage systems.

Despite these advances, current system-level platforms still face several limitations. First, differences in DNA synthesis methods, experimental workflows, and automation architectures among platforms mean that unified standards for performance evaluation remain lacking. Second, most existing systems remain at the prototype stage and face substantial challenges in throughput, cost efficiency, and long-term operational stability, which limit their readiness for large-scale deployment [76]. Future research should develop unified frameworks for system evaluation, strengthen the co-design of encoding strategies and biochemical channel models, and improve the integration of automated laboratory platforms, microfluidic technologies, and enzymatic DNA synthesis. Such advances could help improve throughput, reduce costs, and enhance the scalability of DNA storage systems.

### Synergy between encoding standards and channel modeling

In DNA storage systems, the representation layer and the channel layer correspond to 2 key aspects: information representation and error propagation. The former focuses on how digital data are mapped into DNA sequences, whereas the biochemical channel layer examines errors and molecular changes associated with DNA synthesis, amplification, storage, and sequencing, as well as their effects on data recovery [77]. Although encoding standards and channel modeling tools address different problems of the workflow, their interaction influences the reliability of data writing and recovery.

JPEG DNA and MESA (as shown in Table 3) are representative tools for information representation and biochemical channel modeling. JPEG DNA provides a structured representation scheme for DNA-based image storage by unifying rules for image compression, nucleotide mapping, and sequence organization [78]. In contrast, MESA simulates substitution, insertion, and deletion errors arising during DNA synthesis, amplification, and sequencing, supporting error modeling and performance evaluation [79]. JPEG DNA defines how image information is represented, whereas MESA models how biochemical processes can affect the resulting DNA data. Together, these tools illustrate the complementary roles of information representation and biochemical channel modeling in evaluating DNA storage methods.

**Table 3.** Functional comparison between JPEG DNA and MESA

Category	JPEG DNA	MESA
Tool type	DNA image encoding standard	DNA storage channel simulation and error modeling platform
Research level	Encoding and information representation	Channel modeling and error analysis
Primary objective	Establish a standardized representation from image data to DNA sequences	Model and predict error behavior in DNA storage channels
Main functions	Image partitioning, quaternary nucleotide mapping, syntax specification, and decoding rules	Error modeling during synthesis, amplification, storage, and sequencing (substitutions, insertions, and deletions)
Input/output	Image → quaternary DNA sequence	DNA sequence → predicted error distributions and feasibility evaluation
Methodological role	Ensure encoding consistency and cross-platform interoperability	Evaluate the robustness of coding schemes prior to experimental implementation
Typical applications	Standardized design of DNA-based image storage	Channel prediction and parameter optimization during encoding design
Technical importance	Promote the standardization of DNA image storage	Bridge encoding design and realistic biochemical processes

Unified encoding rules facilitate comparisons of information representation across storage schemes, while channel models link encoding design to biochemical conditions and help assess how encoding strategies may perform under different error conditions. Nevertheless, most existing channel models rely on simplified statistical assumptions and may not fully capture error dependencies and nonuniform error patterns associated with amplification bias, coverage fluctuations, and complex sequence structures [80]. Meanwhile, existing encoding standards are also often designed for specific data types, which may limit their applicability across different information formats. Therefore, future research should develop error models that better capture realistic biochemical conditions and promote the joint optimization of encoding strategies and channel models, with the aim of improving performance prediction and encoding robustness.

### Methodological differences and extensions of general-purpose encoding and decoding toolchains

The general-purpose encoding and decoding toolchains reviewed in this section pursue 3 types of objectives: storage density, system evaluation, and structured information representation (as shown in Table 4). DNA Fountain prioritizes storage efficiency, the DNA Storage Toolkit emphasizes system evaluation capability, and DNA-Chain focuses on content representation and structural awareness. These tools serve different purposes and should not be treated as interchangeable approaches, but rather as distinct approaches representing different trade-offs among storage density, system evaluation, and content representation capability. Recent standardized comparisons of DNA storage codecs have also identified trade-offs among coding schemes in error tolerance, recovery from strand loss, redundancy overhead, and storage density [72]. However, existing comparisons are still affected by differences in datasets, channel models, experimental conditions, and evaluation metrics. A fully unified framework for evaluating DNA storage codecs remains to be established.

**Table 4.** Functional comparison of representative DNA data storage toolchains

Category	DNA Fountain [11]	DNA Storage Toolkit [85]	DNA-Chain [86]
Tool type	Fountain-code-based DNA encoding/decoding framework	Modular end-to-end DNA storage simulation and evaluation toolkit	DNA coding method for structured image data
Workflow	File compression → LT fountain codes → biochemical constraint screening → sequencing and decoding	Encoding → wet-lab simulation → sequence clustering → trace reconstruction → decoding	Contour extraction → chain-code representation → entropy encoding and error correction → DNA sequence generation
Core algorithms	LT, Reed–Solomon error correction, message-passing decoding	GRU-based sequence-to-sequence wet-lab error simulation, q-gram clustering, SIMD-accelerated sequence alignment	Freeman chain, Huffman encoding, Reed–Solomon error correction
Input/output	File → DNA sequence → reconstructed file	File → simulated DNA storage process → reconstructed file	Image → structured feature sequence → DNA sequence → reconstructed image
Availability	Open-source (GitHub: TeamErlich/dna fountain)	Open-source (GitHub: NUS DNA Storage Toolkit)	Research prototype

LT, Luby transform; GRU, gated recurrent unit; SIMD, single instruction, multiple data

Beyond conventional error-correcting codes, recent studies have further utilized the redundancy relationships among fountain-code packets, together with file type and data content information, to recover corrupted or missing data. Deep learning methods have also been used to reconstruct the original DNA sequence from multiple sequencing reads affected by insertion, deletion, and substitution errors, as well as contamination associated with strand breakage, rearrangement, and error clustering. These studies indicate that data recovery in DNA storage can extend beyond reliance on predefined error-correcting codes by incorporating coding structure, data characteristics, and computational inference to expand the range of recovery strategies for damaged data [81,82].

DNA Fountain [11] is a representative DNA storage strategy based on Luby transform (LT) fountain coding, designed to achieve a high information density while maintaining robust data recovery. The input data are divided into multiple segments, which are combined randomly to generate a large number of droplets carrying different segment combinations and associated address information. After sequencing yields a sufficient number of droplets, fountain-code decoding is used to reconstruct the original data. Its principal advantage lies in approaching the theoretical storage limit while maintaining strong data recovery capability. However, its recovery mechanism relies primarily on the redundancy and decodability of droplets, and its actual recovery performance can still be affected by complex biochemical errors, including insertions, deletions, and uneven sequencing coverage. Consequently, its performance depends to a considerable extent on the specific channel conditions. Related studies have further optimized fountain coding for the sequence constraints and channel characteristics of DNA storage. By adjusting coding strategies and related parameters, these studies have sought to optimize the trade-offs among storage density, coding efficiency, and data recovery capability, indicating that fountain coding still has room for improvement in specific DNA storage scenarios [83].

Advances in DNA synthesis and sequencing have highlighted the limitations of relying on coding-layer redundancy alone to address complex errors, including insertions, deletions, and uneven sequencing coverage. Effective recovery also depends on downstream processing, including read preprocessing, clustering, data reconstruction, and retrieval. Pantheon-DNA integrates encoding, decoding, sequencing-data preprocessing, sequence clustering, and retrieval within a common workflow to support DNA storage data processing [84]. Such integrated workflows illustrate the importance of coordinating coding algorithms with sequencing-data processing, recovery, and retrieval.

In contrast, the DNA Storage Toolkit [85] is an open-source, modular end-to-end codec and simulator for DNA data storage research and development rather than for optimizing a specific codec’s efficiency. It integrates encoding, error simulation, sequence clustering, trace reconstruction, and decoding into a unified framework, providing a consistent environment for evaluating different encoding strategies. Using a common workflow can improve result comparability by reducing differences in test implementation. Nevertheless, since the simulation results still rely on the preset error model, their predictive ability is limited by the accuracy of the model.

DNA-Chain [86] is a structure-aware approach to encoding image information. Its basic idea is to convert contour information in images into chain-code representations, followed by compression and DNA encoding, thereby using a structured representation instead of directly storing the original pixel information. DNA-Chain supports the recovery of encoded contour structures and provides a representation suitable for structure-oriented image retrieval. Its structure-aware representation prioritizes contour information over exact pixel preservation, supporting structure-oriented retrieval and subsequent content analysis. However, introducing structural information also consumes part of the available encoding space, requiring a trade-off between storage density and semantic fidelity. The workflows of DNA Fountain, the DNA Storage Toolkit, and DNA-Chain are shown in Fig. 4.

**Fig. 4.**
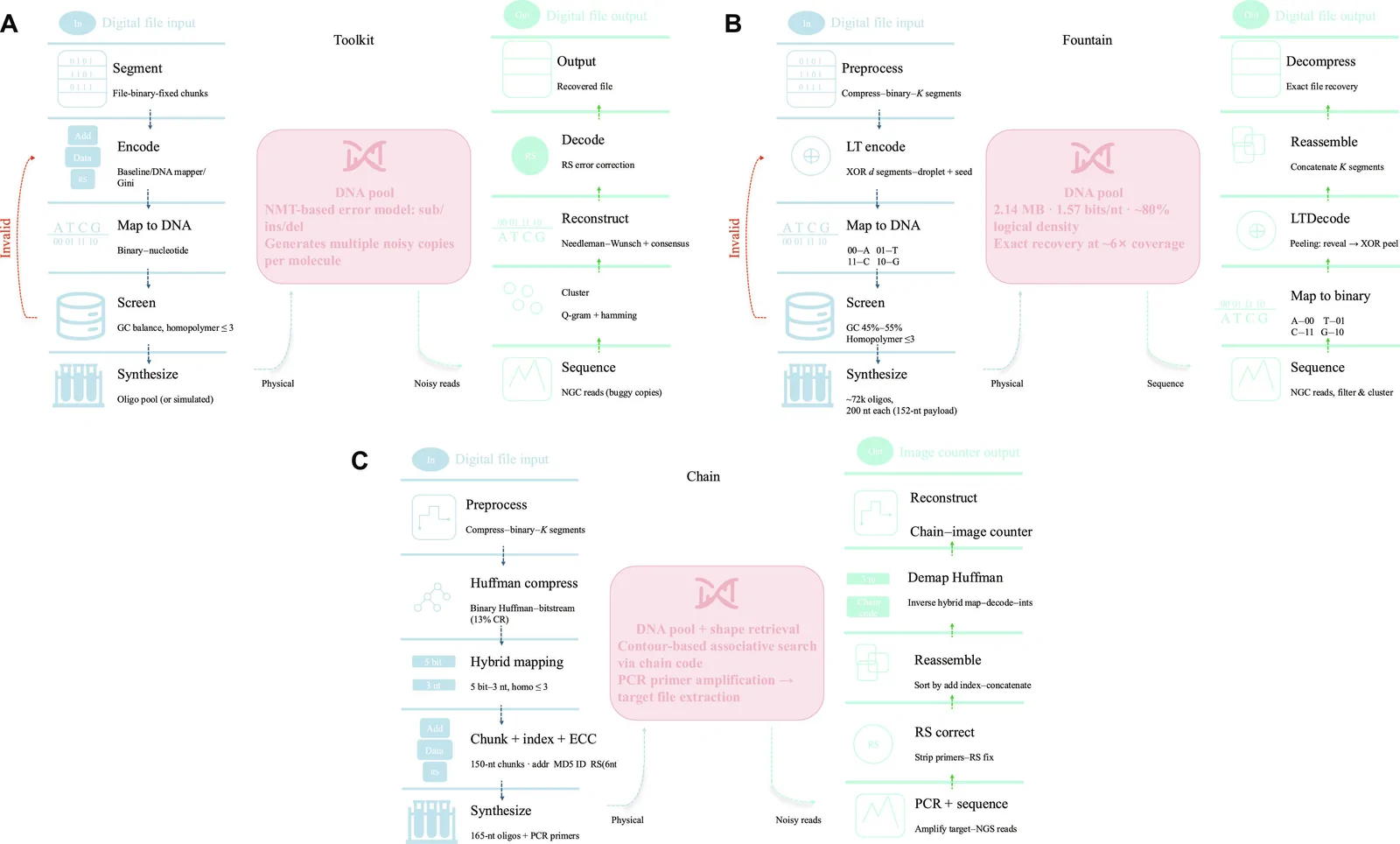
Workflows of 3 representative DNA storage computational tools. (A) DNA Storage Toolkit [85]: modular end-to-end pipeline with 3 interchangeable encoding schemes (baseline/Gini/DNA mapper), a gated recurrent unit (GRU)-based sequence-to-sequence wet-lab simulator modeling sub/ins/del errors, q-gram clustering, Needleman–Wunsch trace reconstruction, and Reed–Solomon decoding. (B) DNA Fountain [11]: Luby transform (LT) fountain-code encoding with random XOR droplets and seed labels, biochemical constraint filtering (GC 45% to 55%, homopolymer ≤3 nt), synthesis of ~72,000 × 200-nt oligos, and LT peeling decoding; the coding potential reaches 1.98 bits/nt under the specified biochemical constraints, while the experimentally achieved effective information density is 1.57 bits/nt. (C) DNA-Chain [86]: structure-aware image encoding via Freeman differential chain code; Huffman compression (13% ratio); hybrid 5-bit-to-3-nt mapping; 165-nt oligos with address, identifier, and Reed–Solomon (RS) error-correction fields; and contour-based associative retrieval; achieves 1.67 bits/nt density with ≥98% recovery at error rates <0.05.

Together, these toolchains illustrate complementary capabilities in storage efficiency, system evaluation and reproducibility, and structure-aware information representation and retrieval [87].

To illustrate the operational characteristics of different DNA information coding methods under their respective task definitions, we selected DNA Fountain, DNA Storage Toolkit, and DNA-Chain as representative case studies; conducted separate tests; and recorded the relevant performance metrics. Table 5 is intended as an illustrative operational case study rather than a controlled head-to-head benchmark or a ranking of method performance. Given their distinct functional positioning—DNA Fountain as a generic file storage codec, DNA Storage Toolkit as a simulation and evaluation platform, and DNA-Chain as a method for structured image data encoding—they were not treated as equivalent encoding tasks. Each method was evaluated within its native encoding framework and under its default parameter settings, using the same computational environment. The quantitative results reported in Table 5 were obtained using one representative image, USC-SIPI 4.2.03 (Mandrill; 512 × 512 RGB TIFF; 786,572 bytes). For each applicable method–channel condition, this image was tested in 10 independent trials using channel seeds 1000 to 1009. The remaining 19 images were subjected to separate recovery-verification runs only and were not included in the quantitative values reported in Table 5. For DNA Fountain and DNA Storage Toolkit, the simulated channels included an error-free E1 channel and an E2 channel with 0.1% nucleotide substitutions and 5% strand dropout. DNA-Chain was evaluated with 0.1% nucleotide substitutions but without strand dropout or insertion/deletion errors, limiting the assessment of its robustness to these channel disturbances. Results are reported as mean ± standard deviation, where applicable. Encoding and decoding times were measured separately as wall-clock times for the respective core processes, excluding process-startup overhead; peak memory was recorded as the maximum resident set size of the full process tree sampled at 5-ms intervals. Redundancy was defined as the ratio of total synthesized nucleotides to the theoretical minimum at 2 bits/nt, including overhead from error correction, indexing, and file identifiers. For each applicable method–channel condition, the recovery rate was calculated as the percentage of the 10 trials on the representative image that achieved successful reconstruction of the method-specific target representation. The reported recovery rates indicate successful reconstruction under the tested *in silico* conditions and should not be interpreted as evidence of equivalent reliability across the 3 methods. All tests were run on an Intel i7-6700 processor with 63.7 GiB RAM under Windows 11 (build 26200), using Python 3.12.4 with fixed dependency versions; all source files were verified using SHA-256 hashes, with full environment details provided in the supplementary file environment.txt. The methods differ in task definition, information representation, and reconstruction requirements. Detailed performance metrics are summarized in Table 5. All results were obtained through computational encoding, channel simulation, and decoding; no physical DNA synthesis or sequencing experiments were performed.

**Table 5.** Illustrative operational case study of DNA Fountain, DNA Storage Toolkit, and DNA-Chain under their respective task definitions. Notes: All quantitative results are based on one representative image, USC-SIPI 4.2.03 (Mandrill; 512 × 512 RGB TIFF; 786,572 B). For each applicable method–channel condition, 10 independent runs were performed using channel seeds 1000 to 1009; results are reported as mean ± SD where applicable. Encoding is deterministic. The remaining 19 images in the 20-image dataset were used only for separate recovery-verification runs and excluded from the quantitative results in this table. Recovery rate is the percentage of the 10 runs achieving exact reconstruction of the method-specific target representation. Encode/Decode are wall-clock times (s), excluding measured process-startup overhead; RSS values are peak process-tree memory (MB), sampled at 5-ms intervals. Redundancy is information-carrying nucleotides (payload plus seed, ECC, index, and identifier fields; PCR/sequencing primer and adapter nucleotides excluded) divided by the theoretical minimum nucleotide count at 2 bits/nt, including ECC, indices, and file IDs. E1, error-free channel. E2, a nucleotide-substitution rate of 0.1% per nucleotide; for DNA Fountain and DNA Storage Toolkit, E2 additionally includes 5% strand dropout without insertions or deletions (indels), whereas DNA-Chain is evaluated without strand dropout because its error correction is intra-strand only. For DNA-Chain, the payload is the in-DNA Huffman-compressed chain-code stream; 66,868 B of reconstruction metadata is stored out-of-band and excluded from synthesized DNA. All oligonucleotide and nucleotide counts refer to computationally designed sequences, not physically synthesized molecules.

Scheme	Oligos	Total nt	Redundancy	Encode	Decode E1	Encode RSS	Decode RSS	Recovery E1	Recovery E2
DNA Fountain	49,162	5,899,440	1.875×	6.10 ± 0.10	24.55 ± 0.19	104.6 ± 0.2	254.0 ± 1.4	100% (10/10)	100% (10/10)
DNA Storage Toolkit	65,535	7,864,200	2.500×	36.47 ± 0.11	46.07 ± 0.12	12.6 ± 0.0	18.0 ± 0.0	100% (10/10)	100% (10/10)
DNA-Chain	685	143,850	1.682×	0.08 ± 0.00	0.15 ± 0.00	56.4 ± 0.4	—	100% (10/10)	100% (10/10)

Oligos, oligonucleotide count; nt, nucleotides; RSS, resident set size; ECC, error-correcting code; PCR, polymerase chain reaction; —, decoding RSS not measured

The test images were obtained from the Miscellaneous (misc) volume of the image database maintained by the University of Southern California Signal and Image Processing Institute (USC-SIPI). This volume contains 39 candidate images, all in TIFF format, including 24-bit RGB images and 8-bit grayscale images. Twenty images were randomly selected from these 39 candidate images to form the test set, comprising 9 RGB images and 11 grayscale images. The images have pixel dimensions of 256 × 256, 512 × 512, and 1,024 × 1,024, with original file sizes ranging from 65,670 to 1,048,710 bytes. The specific IDs of the 20 test images, together with the associated experimental code and configuration files, have been publicly released in a GitHub repository.

Before encoding, the images were processed according to the input requirements of each method. DNA Fountain and DNA Storage Toolkit directly processed the original TIFF file bytes without image-specific preprocessing. DNA-Chain converted each image to grayscale, applied binary thresholding at 127, extracted the contours, and encoded their 8-direction Freeman chain codes as a Huffman-compressed payload. Following the tool’s timing convention, this preprocessing was excluded from DNA-Chain’s reported encoding time. For the separate recovery-verification runs, the remaining 19 images were processed using each method’s corresponding input representation and encoding–decoding pipeline. Reconstruction was checked against the target representation defined by each method: the original TIFF file bytes for DNA Fountain and DNA Storage Toolkit and the thresholded contour representation for DNA-Chain. Because this preprocessing is irreversible, successful reconstruction of the DNA-Chain target representation does not imply recovery of the original image’s complete information content. The same 20 source images were used for all methods, although their input representations differed according to their respective task definitions.

All 3 methods achieved full recovery of their respective output representations under the tested conditions. DNA Fountain and DNA Storage Toolkit target lossless recovery of general binary files, whereas DNA-Chain uses contour chain codes to represent images in a structured manner. Its recovery results primarily reflect the contour information of binarized images. Although DNA-Chain can accurately recover the chain codes and corresponding binary images, grayscale, color, and other information in the original images are discarded during encoding; its recovery capability is therefore not equivalent to lossless recovery of general binary files. In addition, the E2 experiment for DNA-Chain did not use the same 5% strand dropout condition as the E2 experiments for DNA Fountain and DNA Storage Toolkit. For DNA-Chain, the reported total nucleotide count and redundancy exclude out-of-band reconstruction metadata and therefore do not reflect the full storage overhead.

### User-oriented storage and information management platforms

Research on DNA data storage has increasingly addressed not only fundamental challenges in information encoding and data recovery but also data management and application services. Some emerging platforms integrate encoding, decoding, and error evaluation with functions such as database management, sequence indexing, content retrieval, and interactive visualization, thereby establishing a unified interface between biochemical storage processes and end users.

One example is iDNAdrive [19]. Unlike conventional sequence-based DNA storage systems, which typically require DNA resynthesis to modify stored information, iDNAdrive uses DNA modification patterns as the information carrier. By regulating modification patterns at specific loci, it enables data writing, retrieval, and preliminary copying and random access without altering the underlying DNA sequence.

Another research direction is the organization and retrieval of stored content. HELIX [88], designed for biomedical image storage, provides a unified storage framework integrating image compression, DNA encoding, error recovery, and content access. Compared with approaches designed primarily for file-level reconstruction, HELIX uses indexing-free error-correcting encoding and deep-learning-based image repair to reconstruct images with partially missing blocks.

The 2 platforms address different aspects of information management: iDNAdrive supports molecular-level data updating and retrieval, whereas HELIX integrates image compression, DNA encoding, error recovery, and content access. Together, they illustrate complementary approaches to static archival storage with molecular-level readout and content-oriented data organization.

## Computational Framework and Key Challenges in DNA Data Storage

Current research on DNA data storage has covered multiple areas, including information encoding, error control, random access, automated read–write, and data retrieval [89]. However, different studies often focus on specific components of the storage system, with limited attention to their functional roles, internal relationships, and overall development trends. Compared with existing reviews and system architectures for DNA storage, the 5-layer framework proposed in this paper does not reclassify existing methods according to a single technical workflow. Instead, it provides a unified organization of the computational issues in digital DNA storage systems across 5 interconnected functional layers: information representation, encoding and decoding, biochemical channel, system implementation, and access and management. Existing reviews generally focus on the development trajectory of DNA storage technologies, encoding and error-correction methods, and synthesis and sequencing techniques. They are often organized around sequential workflows or specific technological approaches, with relatively limited attention to a functional-layer perspective that connects information representation, encoding and decoding, molecular channels, systems engineering, and data access and management within a unified analytical framework. Taking the 6 main steps summarized by Doricchi *et al.* [23]—encoding, data writing, storage, random access, data readout, and decoding—as an example, their approach organizes DNA data storage according to the sequence of operations, whereas the framework proposed here examines the functional roles, interdependencies, and constraints of different technical components across 5 interconnected functional layers. Our framework therefore emphasizes the functional connections and mutual constraints among different technical components and further incorporates retrieval and access management into a holistic computational perspective on DNA storage systems. It provides a unified basis for comparing technical approaches and examining their functional relationships across the 5 layers.

DNA data storage is not merely a problem of data encoding and decoding but also one of coordinated design across multiple layers including information representation, biochemical channel, system implementation, and data management [87,90,91], creating a need for a unified analytical framework to examine the computational models and technical challenges associated with these functions.

### Functional-layer framework for DNA data storage systems

Existing research on DNA storage covers diverse aspects of the storage workflow, including encoding design, error control, system implementation, and data management. However, these studies focus on different stages and functions of the storage workflow, and a unified basis for comparing their contributions is still lacking [56,76,87]. To clarify the roles of existing tools and platforms in DNA data storage systems, we abstract the storage workflow into 5 interconnected functional layers: the information representation layer, the encoding and decoding layer, the biochemical channel layer, the system implementation layer, and the access and management layer.

The information representation layer is responsible for mapping digital information onto molecular representations in DNA. These representations include conventional sequence encoding and non-sequence-based approaches that exploit molecular features such as spatial structures, topological states, dynamic states, and chemical or epigenetic modifications [4,41,90]. The encoding and decoding layer is responsible for redundancy generation, constrained coding, and data reconstruction, providing essential mechanisms for reliable data storage and recovery [11,72,92]. The biochemical channel layer describes the error mechanisms and their statistical characteristics arising during DNA synthesis, amplification, storage, and sequencing [34,79,80]. The system implementation layer addresses engineering challenges such as automated writing, random access, and end-to-end system integration [12,73,74]. Finally, the access and management layer supports higher-level functions, including data organization, retrieval, and maintenance [12,50,68].

This functional-layer framework is not intended to redefine existing research categories, but rather to provide a functional abstraction of DNA data storage systems from the perspective of the information-processing workflow. Within this framework, representative tools and platforms can be mapped to the functional layers according to their primary roles and capabilities. For example, JPEG DNA primarily belongs to the information representation layer [78], whereas DNA Fountain and the DNA Storage Toolkit are mainly associated with the encoding and decoding layer [11,85]. MESA represents the biochemical channel layer [79], whereas DNA-DISK and automated microfluidic platforms correspond to the system implementation layer [74]. iDNAdrive also illustrates functions at the access and management layer [19]. This framework provides a systems-level view of how existing approaches relate to one another within the overall architecture of DNA data storage.

Compared with reviews organized primarily around individual research topics, this paper takes the complete workflow of DNA information processing as its organizing principle, maps diverse studies onto a unified functional hierarchy, and thereby establishes the connections among information representation, encoding and decoding, biochemical channels, system implementation, and information access. The core value of this framework lies not in proposing new technical categories, but in providing a consistent analytical basis for comparing approaches across different research directions and clarifying their roles within DNA storage systems.

### Key challenges across functional layers

The functional-layer framework described above highlights that the challenges confronting DNA storage systems are not confined to a single stage of the workflow but span multiple functional layers, including information representation, encoding and decoding, biochemical channel, system implementation, and data access and management, as shown in Fig. 5. Although different layers address different aspects of the system, they all reflect the trade-offs among storage efficiency, system complexity, and application requirements.

**Fig. 5.**
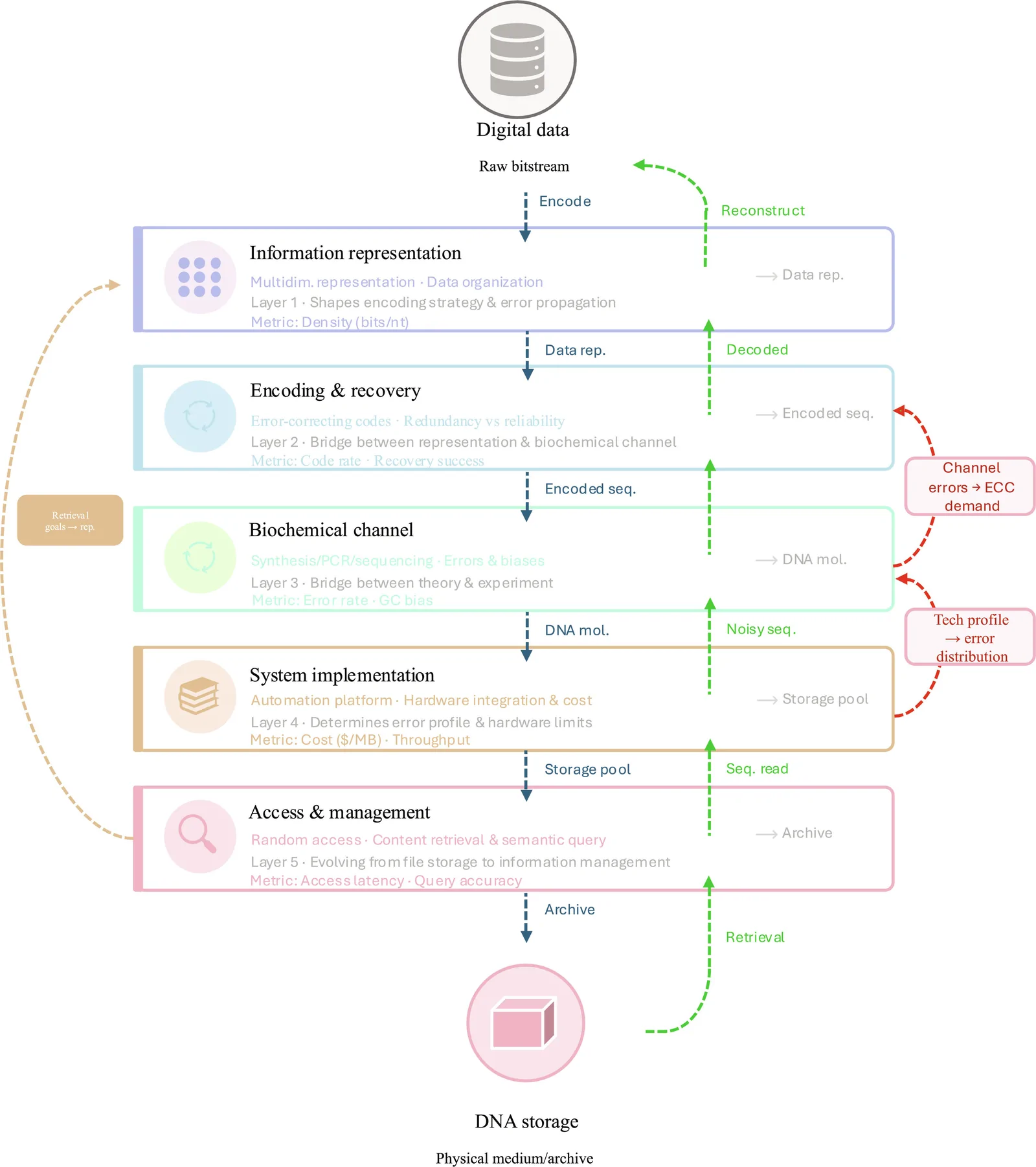
A cross-layer framework of DNA storage systems. The framework comprises 5 interconnected functional layers: (1) information representation, which defines how digital information is represented in DNA (metric: representation-specific information density); (2) encoding and decoding, which introduces redundancy for error correction (metrics: code rate and recovery success); (3) biochemical channel, encompassing synthesis, polymerase chain reaction (PCR) amplification, and sequencing (metrics: per-base error rate and GC-content bias); (4) system implementation, covering automation platforms and hardware integration (metrics: cost per MB and throughput); and (5) access and management, supporting random access, primer design, and content-level retrieval (metrics: access latency and query accuracy).

At the information representation layer, the core issue lies in the balance among information representation capability, storage efficiency, and readout complexity. Sequence-based encoding primarily aims to maximize the amount of information stored per nucleotide, whereas non-sequence-based approaches use molecular features—including spatial conformations, topological configurations, and epigenetic modifications—to represent information. As the information representation space expands, DNA storage may support greater information-carrying capacity, but this expansion also introduces greater molecular diversity, increases the demands on readout and recovery [47], and complicates molecular design and manipulation. Therefore, design choices at the information representation layer must balance the dimensions of information representation, storage efficiency, and readout complexity. Different information-representation methods have distinct error characteristics and biochemical manipulation requirements. Their performance should therefore be evaluated not solely by theoretical storage density but also by considering readout reliability, operational feasibility, and system implementation costs [42,47,86].

At the encoding and decoding layer, the primary challenge is balancing redundancy overhead, recovery reliability, and decoding complexity. Existing approaches—including Reed–Solomon codes and low-density parity-check codes [93]—have improved data recovery capability. However, stronger error-correction capability generally requires additional redundancy, which reduces effective storage density.

Deep-learning-based methods have also been explored for DNA sequence reconstruction, aiming to recover the original reference sequence from noisy sequencing reads [94] while simultaneously satisfying DNA sequence constraints and controlling decoding complexity. In addition, the DNA secondary structure can constrain the biochemical feasibility of designed sequences. Related studies have proposed sequence-design strategies to avoid or minimize potential secondary structures arising from complementary sequences [95]. This further indicates that DNA encoding design must account not only for information density and error-correction capability but also for structural constraints under actual biochemical conditions. As the interface between information representation and the biochemical channel, the encoding and decoding layer depends not only on the encoding and error-correction algorithms but also on the underlying representation scheme and the characteristics of the biochemical error environment. Consequently, the design of different encoding strategies must balance redundancy overhead, recovery performance, and biochemical feasibility.

At the biochemical channel layer, the major challenge lies in the gap between theoretical error models and actual error patterns observed in practice. Errors in DNA storage include substitutions, insertions, and deletions. In addition, PCR amplification bias, GC-content variation, fluctuations in sequencing coverage, and secondary structures can introduce nonuniformities or otherwise affect data recovery [77,96,97]. Therefore, existing idealized error models may not fully capture error distributions under practical storage conditions [72,77]. Meanwhile, DNA information writing itself remains subject to limitations imposed by biochemical processes. The speed and fidelity of DNA synthesis, the achievable sequence length, and synthesis cost directly affect the writing throughput, storage scale, and cost of practical deployment. At the same time, DNA synthesis, sequencing, and related sample processing require chemical reagents, specialized equipment, and energy inputs. Therefore, the energy-efficiency advantage of DNA storage should not be evaluated solely on the basis of the storage medium but should instead be assessed across the relevant stages of the process, including writing, storage, and readout. When comparing energy consumption with traditional storage technologies, the system boundaries and the energy and material inputs included in the comparison should also be clearly defined for the writing, storage, and readout processes. As sequence length increases, the cumulative effects of synthesis errors and incomplete synthesis can reduce the proportion of full-length products, thereby increasing the burden on subsequent screening, error correction, and sequencing. For non-sequence-based molecular writing strategies that use epigenetic modifications, although information recording does not rely on changes to the DNA base sequence, the writing process still depends on specific biochemical reactions and is therefore subject to limitations in reaction speed, writing fidelity, and operational complexity. These considerations indicate that scaling up DNA storage depends not only on advances in encoding and decoding methods but also on continued improvements in DNA synthesis and other molecular writing technologies in terms of speed, fidelity, scalability, and cost [19,53]. Both accurate channel modeling and advances in DNA writing technologies are important for translating theoretical research into practical systems and jointly affect the overall performance and practical applicability of DNA storage systems.

From a commercial perspective, DNA storage still lacks well-defined application scenarios that justify its adoption over conventional storage technologies. Although its high storage density and long-term preservation capabilities offer potential advantages over conventional storage media, these benefits have not yet been translated into clearly defined application scenarios with specific requirements and boundaries. For tasks requiring high-frequency access or real-time read–write operations, DNA storage has a different application profile from mature conventional storage technologies [87,98]. Therefore, further commercialization of DNA storage depends not only on continued improvements in technical performance but also on identifying data types and application scenarios that can make effective use of the advantages of molecular storage and on developing appropriate system-level evaluation criteria.

At the system implementation layer, the main challenge is balancing automation capabilities with engineering costs. Although automated platforms such as DNA-DISK have demonstrated the feasibility of end-to-end DNA storage, the costs of DNA synthesis and sequencing, together with the resource and engineering requirements of automation equipment, continue to constrain further system scaling. Meanwhile, substantial dependencies exist between experimental stages, and performance improvements at a single stage do not necessarily translate into gains in overall system efficiency. Therefore, current DNA storage systems must balance the level of workflow automation, processing throughput, operating costs, and system scalability.

The access and management layer is still at an early stage of development. Traditional DNA storage systems primarily rely on PCR primers to support file-level random access based on file identifiers. Imburgia *et al.* [91] demonstrated the feasibility of extending DNA storage retrieval beyond conventional file-level random access to content-driven semantic search. However, this approach remains at the laboratory-prototype stage and has not yet been deployed at scale. As the volume and variety of stored data continue to grow, relying solely on file identifiers to locate data increases the complexity of index design and retrieval and limits content-centric data organization. Consequently, a principal limitation of the current access and management layer is the lack of mechanisms that directly link stored objects to their content. This limits the transition from file-level random access to finer-grained information access.

From the perspective of practical deployment, the main bottlenecks to scaling up DNA storage lie in molecular writing, data reading, and error recovery. The cost, speed, and scalability of molecular writing are currently among the most immediate constraints. Data-reading efficiency is another key limitation, while errors and recovery requirements affect writing, reading, and data reconstruction throughout the workflow. Previous studies have indicated that the cost, speed, fidelity, and achievable sequence length of DNA synthesis remain important factors constraining DNA storage scale-up, directly affecting writing throughput and the economic feasibility of the system [66,67]. With the development of high-throughput synthesis technologies, research efforts have broadened beyond synthesis efficiency to include reducing the cost per base, improving synthesis fidelity, and increasing the scale of synthesizable sequences. The readout stage is constrained by sequencing cost, speed, and coverage. Sequencing errors, including substitutions, insertions, and deletions, further affect the efficiency and reliability of data reconstruction [51,92]. In response to these limitations, recent data recovery research has explored approaches that extend beyond conventional methods based on predefined error-correcting codes. These approaches may incorporate sequencing-data preprocessing, sequence clustering and reconstruction, redundancy relationships among fountain-code packets, file-content information, or deep learning models [81,82,84]. Collectively, these studies illustrate efforts to improve recovery under complex error conditions by exploiting data characteristics and computational inference. Meanwhile, DNA coding research addresses not only storage density and error-correction capability but also issues such as fountain-code optimization and biochemical constraints on DNA sequences [83,95]. These design considerations require balancing error-correction capability, redundancy overhead, decoding complexity, and biochemical feasibility. Recent comparative studies of DNA storage codecs further indicate that the performance of different approaches is affected by factors such as error types, sequencing depth, physical redundancy, sequence length, and evaluation conditions [72]. Therefore, a key requirement for the practical deployment of DNA storage is to overcome efficiency and cost constraints in molecular writing and readout while reducing data recovery overhead under complex error conditions.

Overall, the aforementioned problems are not independent of one another; rather, they jointly constitute the core challenges in the development of DNA storage systems. The functional layers are closely interrelated: information representation approaches shape the coding strategies and readout mechanisms, the characteristics of the biochemical channel further constrain encoding and decoding performance, and the system implementation and access and management layers determine whether these technologies can be translated into scalable data storage and utilization capabilities. Therefore, DNA storage performance should be evaluated not only in terms of layer-specific metrics but also in terms of considering interactions among the functional layers at the system level [84,91,99].

### From data storage to information systems: Emerging trends

Recent developments in DNA storage increasingly emphasize information organization and utilization, alongside data preservation (Table 6). Early studies primarily focused on demonstrating that digital information could be written into DNA and recovered accurately. Subsequent research increasingly addressed coding efficiency, error-correction capability, and system reliability. More recently, non-sequence-based DNA storage and content-aware encoding have broadened the scope of research beyond conventional sequence-based data representation to include alternative molecular information representations and content-oriented access strategies [90,100,101].

**Table 6.** Major open challenges in DNA data storage

Functional layer	Current progress	Open challenges
Information representation	High-density sequence-based encoding; non-sequence-based encoding	Unified representation of multidimensional information
Encoding and decoding	High-performance error-correction schemes	Content-aware encoding
Biochemical channels	Fundamental error models	Modeling realistic biochemical environments
System implementation	Automated prototype platforms	High-throughput, cost-effective systems
Access and management	File-level random access	Content-level retrieval and database management

This broader research scope does not diminish the importance of conventional sequence-based encoding and reliable recovery; rather, it highlights the need to optimize performance across interconnected functional layers rather than individual metrics in isolation. Recent studies have begun to simultaneously address issues, including sequence design, stability, reliability, scalability, and data access and management, and to enhance overall storage capability through multimodel physical analysis, selective access, and end-to-end system design [84,99,102]. Meanwhile, key challenges in DNA data management remain, particularly in indexing, random access, and repeated read and write operations at scale [100]. Addressing these challenges will require coordinated improvements in more efficient indexing strategies, reliable random-access mechanisms, and support for repeated read and write operations at scale.

## Conclusions and Future Perspectives

### From sequence encoding to multidimensional information representation

Future DNA storage research may extend beyond information representation based solely on nucleotide sequences by exploiting the multidimensional features of DNA molecules, including spatial structure, topological states, dynamic states, and chemical and epigenetic modifications. Compared with conventional sequence-based encoding, multidimensional molecular states can offer additional information-representation dimensions and enable molecular representation schemes to be tailored to different types of information.

Therefore, future research could explore a unified information-representation and encoding framework that relates sequences, structures, and molecular modifications, providing a common basis for mapping and organizing different molecular states. The key to this direction lies not simply in increasing the number of encodable states but in ensuring reliable mapping, readout, and error control across different molecular information dimensions while balancing information capacity, molecular manipulation complexity, and readout reliability.

### From data recovery to content-based/semantic retrieval

Current research on DNA storage primarily focuses on whether data can be accurately recovered, and its evaluation metrics typically include coding efficiency, recovery rate, random-access capability, and error-correction performance. Many DNA storage systems are designed for long-term digital data archiving, with the core objective of reconstructing the original data after the DNA molecules have undergone biochemical processes such as synthesis, amplification, preservation, and sequencing. Therefore, reducing errors and improving data recovery accuracy have long been central research objectives in DNA storage.

As DNA storage datasets grow and access requirements become more demanding, data recovery alone may be insufficient to support efficient information access. For unstructured data such as images, videos, and text, users may need to locate and retrieve relevant content efficiently, whereas conventional file-level random access cannot adequately support content-oriented retrieval.

Building on existing random-access technologies, future research could improve the parallelism and operational stability of DNA data access, develop approaches for scaling up access systems, and establish more effective mappings between physical addresses and information content. At the information organization level, future research could explore vector representations, similarity-based retrieval, and semantic organization, with content features linked to DNA data addresses to support content-based indexing and querying. For unstructured data such as images, it is also possible to investigate the use of deep feature representations and cross-modal retrieval to identify relevant content and link retrieval results to the corresponding DNA data addresses. Furthermore, future research could investigate ways to organize content features, molecular tags, and physical addresses within a common framework, with the aim of supporting content-based indexing and management and providing a basis for selective access as DNA storage systems scale up.

### Cross-layer co-design for realistic biochemical environments

Reliable operation of DNA storage systems under realistic biochemical conditions requires coordinated optimization across information representation, encoding strategies, the biochemical channel, and system implementation. On the one hand, error models should be developed to reflect the characteristics of practical processes, including synthesis, amplification, preservation, and sequencing, so that encoding parameters and error-correction strategies can be optimized to account for the observed error patterns. Meanwhile, coding efficiency, recovery reliability, and decoding complexity should be considered jointly, with the trade-offs between encoding performance and experimental cost tailored to the requirements of different applications. On the other hand, further integration of automated experimental platforms, microfluidic chips, and emerging synthesis and sequencing technologies could support more coordinated experimental workflows. A unified optimization framework could incorporate sample processing, sequencing depth, and data recovery, allowing experimental strategies to be adjusted in response to observed readout results. Such a framework could support the co-optimization of algorithm design and experimental parameters, with the potential to improve system throughput and reduce operating costs.

Furthermore, cross-layer optimization could incorporate feedback-driven closed-loop control, enabling DNA storage systems to continuously adjust their encoding, readout, and recovery strategies based on experimental outcomes. Such approaches could lay the groundwork for coordinating information encoding, molecular manipulation, data readout, and recovery within a common optimization framework, with the aim of improving overall system efficiency and reliability under realistic biochemical conditions.

### From a storage medium to an integrated DNA information system

Future DNA storage systems will need to address not only storage density but also data accessibility, manageability, and the potential integration of molecular-level information processing.

At the data management layer, hierarchical indexing could organize DNA data addresses, molecular tags, and content features within a common framework, supporting information access at different levels of granularity. For large-scale DNA datasets, future systems will need to address data updates, redundancy management, and sample life cycle management while supporting more sustained management beyond one-time writing and bulk recovery.

At the information-processing layer, DNA storage could be combined with molecular recognition and computing mechanisms to explore whether selected screening, matching, and computational tasks can be performed at the molecular level. Compared with approaches that rely entirely on postsequencing computation, molecular-level processing could move selected data screening and matching tasks to the molecular manipulation stage, potentially reducing the amount of data that must be read out and transmitted. The long-term goal is to coordinate information representation, data management, and molecular processing within an integrated system while clearly defining which operations are performed at the molecular level and which require downstream computational processing.

## Data Availability

All data analyzed in this study were derived from publicly available sources. C. Ge is a co-author of DNA-Chain, one of the 3 methods evaluated in the benchmark reported in the “Methodological differences and extensions of general-purpose encoding and decoding toolchains” section. This relationship was managed as follows: The implementation of DNA-Chain used in this case study was written independently by J. Luo, who was not involved in the original DNA-Chain study. It was based solely on the published algorithm and methodological description; no code from the original DNA-Chain implementation was used. The evaluation protocol, workloads, and performance metrics were fixed prior to execution. C. Ge did not execute the benchmark runs and did not perform the primary analysis of the timing or memory measurements. All benchmarking code, configuration files, raw outputs, and detailed environment specifications are available in the public GitHub repository (https://github.com/Chihiro77-love/Table5-performance) to facilitate independent verification. The authors declare no other competing interests.
