# Supplementary material for: Computational Frameworks, System-Level Platforms, and Emerging Trends in DNA Data Storage
Source: Comput Struct Biotechnol J. 2026 Nov 10;35(1):0254. doi: 10.34133/csbj.0254 (PMC13748625; doi:10.34133/csbj.0254)
Supplement: Supplementary file 1 [file csbj.0254.f1.docx]

**Table S1. Operational classification of representative non-sequence-based DNA information technologies**

**Group I. Enabling or foundational molecular technologies**

| Method / Study | Operational classification | Information-bearing state | Writing / readout mechanism | Demonstrated function | Major structural limitations / error modes | Instrumentation requirements |
| --- | --- | --- | --- | --- | --- | --- |
| DNA tile/lattice — Winfree et al., 1998 | Structural foundation | Spatial arrangement of DNA tiles / periodic lattice configuration | Programmable self-assembly through sticky-end interactions / polyacrylamide gel electrophoresis and AFM | Construction and structural characterization of two-dimensional DNA lattices | Incomplete or irregular lattice assembly; structural tears/defects in lattice sheets | DNA synthesis, thermal annealing, polyacrylamide gel electrophoresis, AFM |
| DNA origami — Rothemund, 2006 | Structural foundation | Spatial configuration / surface pattern | Scaffold–staple self-assembly / AFM imaging | Structural patterning and spatial information representation | Folding/assembly defects, including holes, indentations and structural deformation | DNA synthesis, thermal annealing and AFM |

**Continued Table S1. Operational classification of representative non-sequence-based DNA information technologies**

**Group II. Experimentally demonstrated digital information storage systems**

| Method / Study | Operational classification | Information-bearing state | Writing / readout mechanism | Demonstrated function | Logical capacity / density | Physical density | Major error modes | Durability / rewriting | Instrumentation requirements |
| --- | --- | --- | --- | --- | --- | --- | --- | --- | --- |
| Dynamic DNA — Chandrasekaran et al., 2017 | Rewritable molecular-state memory | Reversible molecular conformational states | Toehold-mediated strand displacement / gel electrophoresis | Writing, erasing, rewriting and molecular logic | 5 bits per memory system | NR | Incomplete erasure and state-transition variability associated with hybridization kinetics, strand competition, loop size and sequence | Rewriting demonstrated; practical upper limit estimated at ~10–100 write/erase cycles | Room-temperature mixing and standard gel electrophoresis |
| Epigenetic molecular storage — Zhang et al., 2024 | Molecular-state information storage | DNA methylation state | Self-assembly-guided enzymatic methylation / nanopore sequencing and methylation calling | Information writing, readout and recovery | 350 bits per writing reaction; ~275,000 bits written | NR | Methylation-calling errors, barcode misalignment/misligation, and writing/readout errors | Thermal stability demonstrated at 65–95 °C for 1 h; rewriting not established as a core demonstrated function | DNA self-assembly, DNMT1-mediated methylation, automated liquid handling, nanopore sequencing and computational methylation calling |

**Note:** NA, not applicable; NR, not reported. The classification is based on the experimentally demonstrated information-bearing state and function of each study. Systems that jointly use DNA sequence and non-sequence molecular states as information-bearing variables are classified as hybrid sequence/non-sequence approaches. DNA tile/lattice and DNA origami studies are treated primarily as structural foundations for non-sequence-based DNA information representation rather than direct digital storage systems. Capacity or density values are listed only when explicitly reported in the original studies and are not inferred from structural dimensions.

Table S2. Summary of representative DNA information storage systems

| Method / Study | Encoding density / Information metric | Redundancy / Error correction | Recovery performance | Synthesis and sequencing | Access method | Experimental scale |
| --- | --- | --- | --- | --- | --- | --- |
| Church et al., 2012 | 1 bit/base; 5.27 Mbit  total ; total capacity | Consensus decoding; no dedicated ECC | 10 bit errors in 5.27 Mbit; all data blocks recovered | Inkjet DNA synthesis; Illumina HiSeq; ~3,000× coverage | Sequential | 54,898 oligos × 159 nt |
| Goldman et al., 2013 | 1.58 bits/nt (encoding potential)  per nucleotide | 4-fold overlap redundancy; address/parity/check sequences | 4/5 files decoded without intervention; the fifth required manual correction of two 25-nt gaps; all five files ultimately recovered with 100% accuracy | DNA microarray synthesis; Illumina HiSeq | Sequential | 757,051 bytes; 153,335 × 117 nt |
| Erlich & Zielinski, 2017 (DNA Fountain) | 1.57 bits/nt  per nucleotide | 7% redundancy; fountain codes + Reed–Solomon coding | Complete recovery with zero errors | Oligo synthesis; Illumina MiSeq | Sequential | 2.14 MB; 72,000 oligos; 152-nt encoding length |

Continued Table S2. Summary of representative DNA information storage systems

| Method / Study | Encoding density / Information metric | Redundancy / Error correction | Recovery performance | Synthesis and sequencing | Access method | Experimental scale |
| --- | --- | --- | --- | --- | --- | --- |
| Organick et al., 2018 | 1.10 bits/nt excluding primers; 0.81 bits/nt including primers | Reed–Solomon coding; primer-based addressing | Individual files recovered without errors | DNA synthesis; Illumina NextSeq; Oxford Nanopore validation | Random access | 35 files; >200 MB; >13 million oligos |
| Anavy et al., 2019 | 1.96 bits/synthesis cycle (large-scale Σ6 system)  per synthesis cycle | Error-correcting schemes + statistical inference | Successful decoding of composite-letter data | Composite DNA synthesis and sequencing | Sequential | ~20 MB of coded data |
| Pan et al., 2022 | NR | Optional systematic LDPC; 3.3% overhead for unequal protection | 11,726 sequences perfectly recovered; 22 contained errors; 78 were highly corrupted or missing | IDT synthesis; Illumina MiSeq | Sequence + nick/topology-based readout | 8 images; 8.65 Mbit; 11,826 oligos |

Continued Table S2. Summary of representative DNA information storage systems

| Method / Study | Encoding density / Information metric | Redundancy / Error correction | Recovery performance | Synthesis and sequencing | Access method | Experimental scale |
| --- | --- | --- | --- | --- | --- | --- |
| Zhang et al., 2024 | 350 bits/reaction; ~275 kbit total  per reaction; total capacity | Compression + error correction | Sequencing retrieval accuracy of 97.5–99.4%; 97.47% in the panda experiment, followed by perfect image restoration after error correction | Synthesis-free enzymatic methylation; nanopore sequencing | Preliminary random-access demonstration | 700 DNA movable types; 5 templates; ~269 kbit in the largest experiment |
| Zhang et al., 2025 (DONLDS) | RDON: 222.22 Gbit/cm²; CDON: 177.76 Gbit/cm²; TDON: 157.78 Gbit/cm²  per unit area | Structural cross-validation | 100% bitwise recovery for 24 randomly selected samples (1,728 bits) | DNA origami nanostructures; AFM readout | Linked-list / structural access | 9-node linked structures; 24 samples / 1,728 bits |
| Ge et al., 2026 (DNA diamond) | 8-letter: 2.5 bits/letter; 15-letter: 3.125 bits/letter  per composite letter | Composite-letter encoding + indexing / length filtering | Error-free at 14× coverage for the 8-letter system and 33× for the 15-letter system | Array-based composite DNA synthesis and sequencing | Indexed sequence readout | 10,000 composite strands per large-scale pool |

NR, not reported. The metric basis or denominator is indicated directly below each reported density or information metric. Information-density values are presented according to the definitions used in the cited studies or subsequent comparative analyses. Because different studies may include or exclude payload, address, index, primer, and other non-payload regions differently, density values should not be interpreted as strictly equivalent across all systems. Values directly reported by the original studies are distinguished from metrics compiled or normalized in subsequent comparative analyses. For composite-letter and structural-storage systems, density is reported using the native metric of the corresponding system (e.g., bits per synthesis cycle, bits per composite letter, or Gbit cm−2) and should not be directly compared with conventional bits-per-nucleotide metrics.
